# MTA1-upregulated EpCAM is associated with metastatic behaviors and poor prognosis in lung cancer

**DOI:** 10.1186/s13046-015-0263-1

**Published:** 2015-12-23

**Authors:** Ning Zhou, Haijuan Wang, Hongxu Liu, Hongsheng Xue, Feng Lin, Xiting Meng, Ailing Liang, Zhilong Zhao, YongJun Liu, Haili Qian

**Affiliations:** Department of Biochemistry and Molecular Biology, Medical Molecular Diagnostics Key Laboratory of Guangdong, Guangdong Medical University, 1 New Town Road, Dongguan, 523808 China; Key Laboratory of Molecular Oncology, Cancer Institute and Hospital, Chinese Academy of Medical Sciences, 17 Panjiayuan Nanli, Beijing, 100021 China; Department of Thoracic Surgery, the First Affiliated Hospital, China Medical University, Shenyang, 110001 China; Department of Thoracic Surgery, the Affiliated Zhongshan Hospital of Dalian University, 6 Jie Fang Road, Dalian, 116001 China; Department of Clinical Biochemistry, Medical Molecular Diagnostics Key Laboratory of Guangdong, Guangdong Medical University, Dongguan, 523808 China

**Keywords:** Lung cancer, MTA1, EpCAM, Invasion, Migration

## Abstract

**Background:**

Overexpression of Metastasis-associated protein 1 (MTA1) in various cancer cells promotes tumor invasion and migration and predicts cancer patients’ poor prognosis. The pilot RNA-Seq data from our laboratory indicated that Epithelial cell adhesion molecule (EpCAM) was statistically reduced in MTA1-silencing cells. EpCAM has been recognized as more than a mere cell adhesion molecule and recent findings have revealed its causal role in mediating migratory and invasive capacity. Thus, this study was aimed to explore whether MTA1 was able to upregulate EpCAM expression and, consequently, modulate its effects on invasion and migration of the lung cancer cells as well as patients’ prognosis.

**Methods:**

We checked the EpCAM expression by overexpressing or silencing MTA1 in lung cancer cells. Furthermore, these lung cancer cells with stably overexpressed or silenced MTA1 were transfected with siEpCAM or EpCAM-expressing plasmids and then subjected to western blot, invasion and migration assays. In addition, patients (*n* = 118) with early-stage lung cancer were enrolled in this study to confirm the correlations between MTA1 and EpCAM and pathoclinical parameters by using immunohistochemistry (IHC). All statistical analyses were performed with SPSS 20.0 statistical software.

**Results:**

MTA1 upregulated EpCAM expression in lung cancer cell lines, and EpCAM overexpression rescued the inhibitory effects by silencing MTA1 on cell invasion and migration in vitro. What’s more, both MTA1 and EpCAM, correlated to each other, were overexpressed in lung cancer tissues and significantly correlated with their clinical stages, tumor diameters, lymph node metastasis. Multivariate analysis indicated that local advancement (*p* = 0.03), MTA1 overexpression (*p* = 0.001) and EpCAM overexpression (*p* = 0.045) of the lung cancer tissues remained significant in predicting unfavorable overall survival.

**Conclusions:**

We revealed a new molecular mechanism of MTA1-mediated invasion and metastasis in lung cancer through downstream target EpCAM, and interfering with EpCAM function may be a novel therapeutic strategy for treatment of MTA1-overexpressing lung carcinoma.

## Background

Lung cancer is one of the most common and deadly tumors in the world. From 1995 to 2009, 5-year overall survival of lung cancer patients remains as low as 20 % in developed and developing countries [1]. Clinical data show that over 90 % of the deaths of patients with lung cancer are direct or indirect consequence of cancer invasion and metastasis [2].

Cancer metastasis is a multi-step process during which cancer cells detach from the primary site, penetrate into the circulation and colonize at distant organs. Many studies have implicated Metastasis-associated gene 1 (MTA1) as a pivotal regulator during cancer metastasis. MTA1 is a subunit of the nuclear remodeling and deacetylation (NuRD) complex, which is over-activated in numerous malignant cancers including lung, breast, and prostate cancers [3–5]. MTA1 not only functions as a co-activator on certain promoters, including β-catenin, BCAS3 and STAT3, but also acts as a transcriptional co-repressor of some target genes, such as E-cadherin [6]. Other than NuRD complex, there are other mechanisms adopted by MTA1 to exert a widespread regulation on target molecules, such as acetylation modification. Despite some targets that have been documented, mechanistic understanding that how MTA1 induces metastasis is still opaque in lung cancer.

Accumulating evidence suggests that MTA1 may promote tumorigenesis and tumor aggressiveness through changing the expression of cell adhesion molecules. Wang et al. identified that MTA1 can facilitate tumor invasion by downregulating E-cadherin in esophageal squamous cell carcinoma [4]. Also, RNA-seq of the MTA1-silenced cells from our laboratory indicated that Epithelial cell adhesion molecule (EpCAM) significantly positively correlated with MTA1 (unpublished results). EpCAM is a transmembrane glycoprotein, which ranges its power from cell-to-cell adhesion to cell proliferation, differentiation and migration [7]. Recently, a growing body of literatures suggests that over-expressed EpCAM facilitates tumor invasion and migration in breast cancer, colorectal cancer and thyroid cancers [8–10]. Based on the results described above, we hypothesized that MTA1 may regulate EpCAM to promote cancer invasion and migration and, consequently, put its effects on the prognosis of lung cancer patients.

In this study, we demonstrated that over-expression of MTA1 induced EpCAM expression in human lung cancer cell lines, leading to increased cell invasion and migration. Expression of MTA1 and EpCAM is strongly correlated to each other in lung cancer tissues and high-level MTA1 and EpCAM predict patients’ poor prognosis in multivariate analysis.

## Methods

### Cell lines and cell culture

Cancer cell lines A549 (adenocarcinoma cells), PC-9 (adenocarcinoma cells), and NCI-H446 (small cell lung cancer cells) cells were maintained in our laboratory. These cells were cultured in RPMI1640 medium supplemented with 10 % fetal bovine serum (FBS), penicillin (100 U/ml), and streptomycin (100 mg/ml). These cells were incubated at 37 °C and 5 % CO_2_ in a humidified atmosphere.

### Establishment of MTA1-knockdown and MTA1-overexpressing cells

A549, PC-9, NCI-H446 cells were transfected with Lenti-shMTA1, Lenti-MTA1, and Lenti-GFP (The three vectors were purchased from Shanghai GenePharma Co, Ltd. (Shanghai, China) at 40 MOI in 24-well culture plate. Puromycin was used to screen the cells positively infected by the vectors. Finally, western blot analysis was performed to examine the MTA1 expression level. The MTA1-over-expressing cells were separately named as A549-MTA1, PC-9-MTA1, and NCI-H446-MTA1 cells, and MTA1-silencing cells were respectively named as A549-shMTA1, PC-9-shMTA1, and NCI-H446-shMTA1 cells.

### The siRNAs and plasmids transfection

MTA1-overexpressing or silenced cells were transfected with EpCAM siRNA or EpCAM-expressing plasmid respectively following the procedures stated in our previous publication [11]. The EpCAM small interfering RNA (siRNA) and negative control were purchased from Shanghai Gene Pharma Co. Ltd (Shanghai, China), and the target sequence of EpCAM siRNA was 5′-UGCCAGUGUACUUCAGUUGTT-3′. The pEnter-C-RFP-EpCAM and pEnter-C-RFP plasmids were bought from ViGene Co, Ltd. (Shandong, China). The GenBank accession number was NM-002354. The MTA1-overexpressing cells were transfected with EpCAM siRNA and NC siRNA, and the MTA1-knocking down cells were transfected with pEnter-C-RFP-EpCAM and pEnter-C-RFP for 24 h before they were harvested for transfection efficiency assay and biological assays.

### Western blot analysis

Cells were lysed in RIPA buffer, and the protein concentration was measured using the Bradford assay. The whole protein samples were loaded onto 10 % SDS-PAGE gels, and were transferred to a PVDF membrane after electrophoresis. The membrane was blocked with 5 % fat-free milk, and then incubated with anti-MTA1 polyclonal antibody (ab71153; Abcam, Cambridge, MA, USA), anti-EpCAM polyclonal antibody (A1177; ABclonal, Boston, MA, USA) and anti-β-actin monoclonal antibody (A5316; Sigma-aldrich, St. Louis, MO, USA) overnight at 4 °C, followed by peroxidase-conjugated secondary antibody (1:5000) incubation for 1 h at room temperature. Signals were visualized with ECL and detected using LAS 4000 Imaging system (Fijifilm, Tokyo, Japan).

### Transwell invasion assay

Cells were seeded in serum-free medium in the upper wells of transwell chambers with a 8-μm pore polycarbonate membrane (#3422; Corning Life Sciences, Tewksbury, MA, USA). The bottom wells were filled with fetal bovine serum-completed medium (600 μL/well). After cells were incubated for 48 h, the cells transmembraned through and adhered to the bottom side of the membrane were fixed and stained with 0.2 % crystal violet dye. Then the cells were counted and averaged in the randomly chosen five fields under microscope.

### Wound healing assay

MTA1-knockdown cells transfected with pEnter-C-RFP-EpCAM (or pEnter-C-RFP), and MTA1-overexpressing cells transfected with siEpCAM (or negative control) were cultured in 12-well plates. When the cells reached 80 % confluence, a sterilized pipette tip was used to scratch a fine line with the same width on the cell layer. The detached cells were washed off with PBS and the remaining cells were cultured in complete medium. Photos were taken at 0, 12 and 24 h after the scratching. Cells motility was presented by measuring the distance of cells moving cross the scratching line under microscopy during the healing of the wound.

### Patients and tissue microarray

The tissue microarray included 118 early stage lung carcinomas (stagesI, II, and IIIA) and 12 non-neoplastic samples (who underwent surgical resection between 1998 and 2000 in the First Affiliated Hospital of China Medical University). All the samples were fixed in formalin, embedded in paraffin, and sectioned at 4-μm for immunohistochemistry and hematoxylin-eosin staining. Patients with stage IIIB or IV lung cancers or incomplete clinical data were excluded from our analysis. The diagnostic and histopathologic classification were assessed according to World Health Organization criteria [12]. The information of clinical pathological variables was collected from the patients’ medical records. This study was approved by the Ethics committee of the First Affiliated Hospital of China Medical University, and informed consent has been signed by every patient. The data of clinical pathological variables were summarized in Table 1.

**Table 1:** Correlation of MTA1 and EpCAM with clinicopathological characteristics of lung cancer patients

Variables	MTA1			EpCAM		
	Low	High	*p*	Low	High	*p*
(*N* = 118)	53	65		51	67	
Age (years)^a^						
> = 57 (65)	28	37	0.66	26	39	0.44
<57 (53)	25	28		25	28	
Gender						
Male (90)	45	45	0.05	42	48	0.20
Female (28)	8	20		9	19	
Histopathologic types						
SCC (35)	21	14	0.02	19	16	0.02
ADC (46)	14	32		14	32	
LCC (8)	3	5		5	3	
SCLC (13)	8	5		9	4	
Others^b^ (16)	6	10		4	12	
Differentiation						
Well (24)	15	9	0.12	11	13	0.85
Moderate (26)	9	17		10	16	
Poorly (68)	29	39		30	38	
Tumor diameter, cm						
<3 (12)	9	3	0.04	6	6	0.78
3–5 (75)	34	41		33	42	
>5 (31)	10	21		12	19	
Local advance						
T1,2 (94)	46	48	0.11	42	52	0.65
T3,4 (24)	7	17		9	15	
Lymph node metastasis						
N0 (53)	35	18	0.01	30	23	0.01
N1,2,3 (65)	18	47		21	44	
Clinical stage						
I/II (71)	43	28	0.01	37	34	0.02
IIIA (47)	10	37		14	33	
Smoking status						
No (45)	13	32	0.01	13	32	0.02
Yes (73)	40	33		38	35	
Neoadjuvant treatment						
NO (36)	21	15	0.07	19	17	0.20
Yes (82)	32	50		32	50	

*SCC* squamous cell carcinoma, *ADC* adenocarcinoma, *LCC* Large cell carcinoma, *SCLC* small cell lung cancer

^a^Age: The median age is 57 years old

^b^Others: adenosquamous carcinoma and salivary-gland carcinoma

### Immunohistochemical staining

The slides were baked at 60 °C for overnight, dewaxed in xylene and rehydrated with gradient alcohol, then the slides were boiled in citrate buffer for antigen retrieval. After that, the slides were immersed in 3 % H_2_O_2_ to inactivate endogenous peroxidase and blocked with goat serum for 30 min. Next, the slides were incubated with the primary monoclonal against MTA1 (ab71153; Abcam, Cambridge, MA, USA) and EpCAM (A1177; ABclonal, Boston, USA) diluted in 1:400 and 1:200 respectively in PBS at 4 °C, followed by incubation with biotinylated-second antibodies and streptavidin-peroxidase complex. And protein staining signal was then observed with 3′-diaminobenzidine. Lastly, the slides were counterstained with hematoxylin and mounted with neutral balsam. For a negative control, the slides were incubated with PBS instead of the primary antibody.

### Scoring system and cutoff points

The IHC staining of MTA1 and EpCAM in the slides was evaluated in a double-blinded manner by using a semi-quantitative immune reactivity scoring system (IRS) for both staining intensity (0: negative; 1: weak; 2: moderate; 3: intense) and staining percentage of positively stained cancer cells (0: none; 1: <10 %; 2: 10–50 %; 3: 51–80 %; 4: >80 %). The final staining scores ranged from 0 to 12. A score >4 was defined as strong MTA1 and EpCAM expression, and a score ≤4 was defined as negative or weak expression of MTA1 and EpCAM [13, 14].

### Statistical analysis

All statistical analyses were performed using SPSS 20.0 software. A Student *t*-test or ANOVA test was used for comparison of quantitative data. The *χ*^2^ test was used to examine the correlation of MTA1 or EpCAM with patients’ clinicopathological factors. The association between MTA1 and EpCAM was analyzed by nonparametric test (Spearman test). Survival rates were determined using the Kaplan-Meier method and were analyzed using the log-rank test. The prognostic significance of clinicopathological variables was also identified by univariate and multivariate analysis using Cox proportional hazard regression model. The statistical significance was considered when the *p* value was <0.05.

## Results

### MTA1 upregulated EpCAM expression in lung cancer cells

**Fig. 1:**
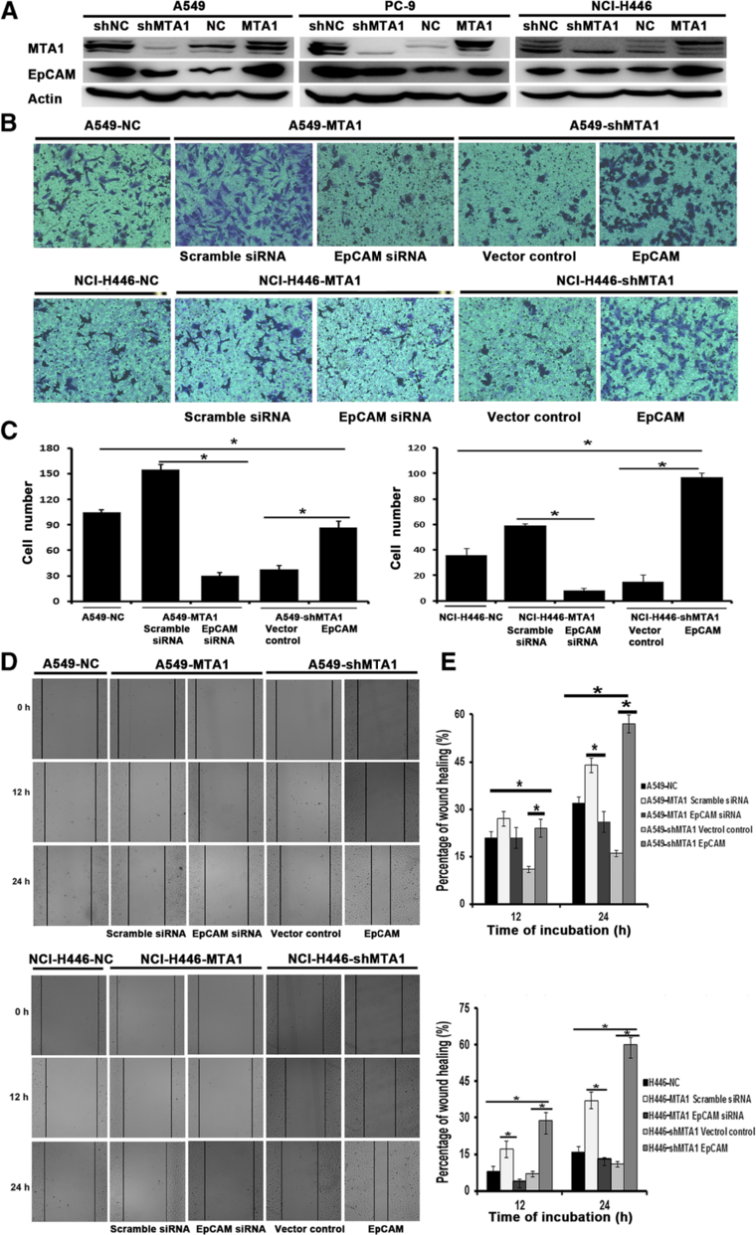
MTA1 could upregulate EpCAM expression and promote invasion and migration via upregulation of EpCAM expression in lung cancer cells. **a** Western blot analysis of the expression level of EpCAM in MTA1-overexpressing or MTA1-silencing cells. shNC: A549, PC-9, NCI-H446 cells transfected with Lenti-shNC-GFP; shMTA1: A549, PC-9, NCI-H446 cells transfected with Lenti-shMTA1; NC: A549, PC-9, NCI-H446 cells transfected with Lenti-NC-GFP. MTA1: A549, PC-9, NCI-H446 cells transfected with Lenti-MTA1. **b** Images of the transwell invasion assay in NC cells, MTA1-overepressed cells transfected with Scramble siRNA or EpCAMsiRNA and MTA1-silenced cells transfected with vector control or EpCAM. **c** Quantitative analysis of the number of invasion cells in the five groups of cells. The invaded cells were counted under × 200 magnification (five randomly selected fields). First, the number of invasive shMTA1 cells was significantly reduced (*p < 0.05, shMTA1 vs. shNC), and the number of invasive MTA1 cells was dramatically increased (*p < 0.05, MTA1 vs. NC). Second, invasive response of MTA1-overexpression cells was impaired significantly after downregulatingEpCAM (*p < 0.05, EpCAMsiRNA vs. Scramble siRNA in MTA1-overexpression cells), and invasion could be rescued by overexpressing EpCAM (*p < 0.05, EpCAM vs. Vector control in MTA1-silencing cells). **d** Representative images of the five cell groups in the wound healing assay (magnification,×200). **e** Quantitative analysis of the migration ability of lung cancer cells. First, the MTA1-silencing could reduce cells motility when compared with the negative controls cells (*p < 0.05, shMTA1 vs. shNC), and the MTA1-overexpression could increase cells motility when compared with the negative controls cells (*p < 0.05, MTA1 vs. NC). Second, a knockdown of EpCAM expression in MTA1-overexpression cells could dramatically attenuate cell migration (*p < 0.05, EpCAMsiRNA vs. Scramble siRNA in MTA1-overexpression cells), and the upregulation of EpCAM in MTA1-silencing cells could regain cell migration (*p < 0.05, EpCAM vs. Vector control in MTA1-silencing cells). All experiments were repeated three times

To determine whether MTA1 can induce EpCAM expression in lung cancer cells, Lenti-shMTA1 and Lenti-MTA1 were used to stably infect the three lung cancer lines (A549, PC-9 and NCI-H446). In this context, overexpression of MTA1 in the three lung cancer cell lines increased EpCAM expression at protein level (Fig. 1a). Conversely, MTA1 knockdown in the three lung cancer cell lines suppressed EpCAM protein expression (Fig. 1a).

### EpCAM induction was critical for MTA1-mediated cell invasion and migration

MTA1 has been suggested to be involved in cell invasion and migration. To confirm whether EpCAM mediates the effects of MTA1 on cell invasion and migration, we conducted the transwell invasion assay and wound healing assay in lung cancer cells. First, using A549 and NCI-H446 cells infected with Lenti-shMTA1, Lenti-MTA1, and Lenti-GFP, we performed transwell invasion assay and found that MTA1-overexpressing cells were significantly more invasive than control cells (155 ± 5.7 vs.105 ± 2.5 in A549 control cells, and 59 ± 1.4 vs. 36 ± 4.9 in NCI-H446 control cells, *p* < 0.05, Fig. 1b, c), and MTA1-silenced cells was significantly impaired for their invasion potential compared with negative control cells (52 ± 5 vs.105 ± 2.5 in A549 control cells, and 15 ± 5 vs. 36 ± 4.9 in NCI-H446 control cells, *p* < 0.05, Fig. 1b, c). Secondly, EpCAM siRNA and scramble siRNA were transfected into MTA1-overexpressing cells (A549-MTA1 and NCI-H446-MTA1), and pEnter-C-RFP-EpCAM (RFP-EpCAM) and pEnter-C-RFP (RFP-NC) were transfected into MTA1-knockdown cells (A549-shMTA1 and NCI-H446-shMTA1) to see the mutual function rescue and abolition effects . We found that invasion potential of MTA1-overexpressing cells was decreased significantly (155 ± 5.7 vs. 30 ± 3.8in A549-MTA1 control cells, and 59 ± 1.4 vs. 8 ± 1.7 in NCI-H446-MTA1 control cells, *p* < 0.05, Fig. 1b, c) after reducing EpCAM level, and the invasion ability in MTA1-silenced cells could be rescued by overexpressing EpCAM (52 ± 5.0 vs. 123 ± 7.5in A549-shMTA1 cells, and 15 ± 5.0 vs. 97 ± 3.0in NCI-H446-shMTA1 control cells, *p* < 0.05, Fig. 1b, c). Third, we also performed wound healing assay using the same cell set. Lenti-shMTA1 infection inhibited cell migration, and the Lenti-MTA1 infection promoted migration capacity of cancer cells when compared with negative control cells (*p* < 0.05, Fig. 1d, e). Similarily, reduced EpCAM expression in MTA1-overexpressing cells dramatically impaired cell motility, and upregulation of EpCAM in MTA1-silencing cells restored cell motility (*p* < 0.05, Fig. 1d, e). Taken together, these results indicate that MTA1 signals through EpCAM, at least in part, to stimulate the invasion and migration abilities in lung cancer cells.

### Expression of MTA1 and EpCAM in lung cancer and non-neoplastic lung tissues

**Fig. 2:**
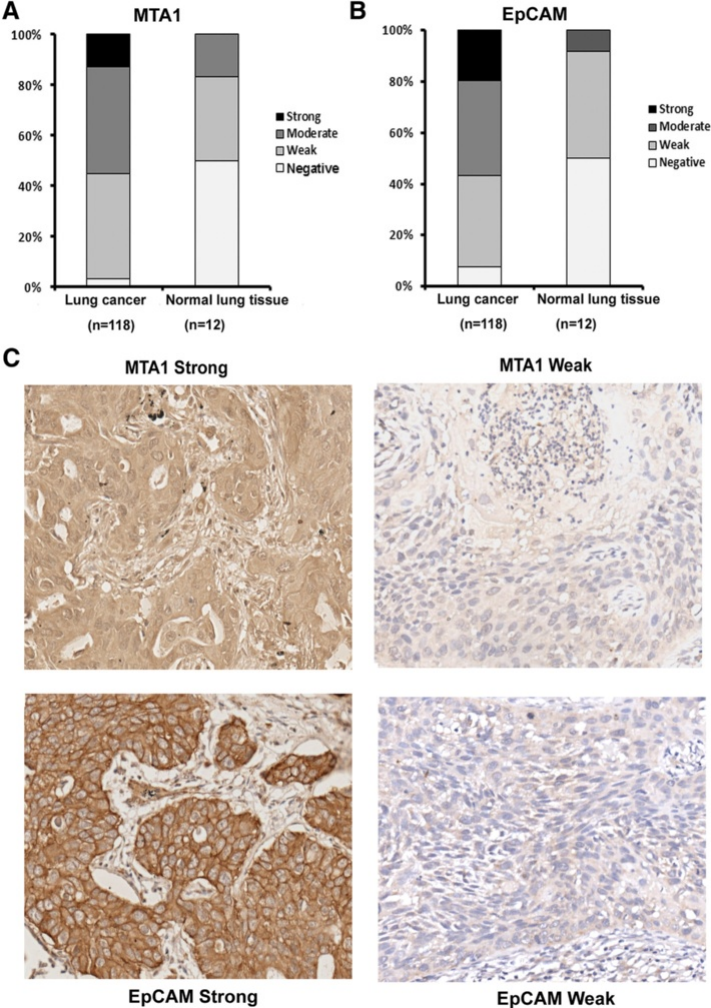
Expression of MTA1 and EpCAM in tissue microarray samples. **a** The immunohistochemistry results showed that MTA1 were overexpressed in lung cancer tissues when compared with nonneoplastic tissues (*p < 0.05).**b** The immunohistochemistry results showed that EpCAM were overexpressed in lung cancer tissues when compared with nonneoplastic tissues (*p < 0.05). **c** Representative images showing immunohistochemical expression in lung cancer tissue specimens of MTA1 and EpCAM. Cases with strong and weak expression are represented. Original magnification, ×200

Of the 118 early stage lung cancer specimens, a high level of MTA1 was detected in 65 (55.10 %) cancer tissues, but only in 2 (16.70 %) of 12 non-neoplastic lung tissues (Fig. 2a). The staining of MTA1 protein exhibited both nuclear and cytoplasmic location in the lung cancer tissues (Fig. 2c). For EpCAM protein, it was overexpressed in 67 (56.8 %) cancer tissues, but only in 1 (8.3 %) nonneoplastic lung tissues (Fig. 2b). In positively stained cancer cells, EpCAM was primarily localized on the cell membrane, with occasional diffusion in cytoplasm (Fig. 2c).

### Correlation of MTA1 expression with clinicopathological variables

Table 1 showed the correlations of MTA1 expression with the clinicopathological variables in lung cancer cases. Significantly positive associations were identified between MTA1 expression and tumor diameter (*p* = 0.04), lymph node metastasis (*p* < 0.01), clinical stage (*p* < 0.01), but negative correlation was found with smoking status (*p* = 0.01). A significant difference was also proved for MTA1 overexpression in cancers of various histopathological subtypes (*p* = 0.03). Further analysis found that the frequency of MTA1 overexpression was lower in squamous cell carcinoma (SCC) and small cell lung cancer (SCLC) than in adenocarcinoma (ADC). However, no significant correlation was found between MTA1 expression and age, gender, differentiation, local advance and neoadjuvant treatment (*p* > 0.05).

### Correlation of EpCAM expression with clinicopathological variables

Table 1 also summarized the correlation of EpCAM expression and clinicopathological variables in lung cancer cases. EpCAM overexpression was significantly positively associated with lymph node metastasis (*p* = 0.01), clinical stage (*p* = 0.02), while negatively associated with smoking status (*p* = 0.02). Similar to MTA1, a significant difference in EpCAM expression was found between various histopathological subtypes (*p* = 0.02). ADC had a higher EpCAM overexpression rates compared to other histopathological subtypes. But no significant correlation were observed for EpCAM in relation to other clinicopathological characteristics.

### Correlation between MTA1 and EpCAM

**Fig. 3:**
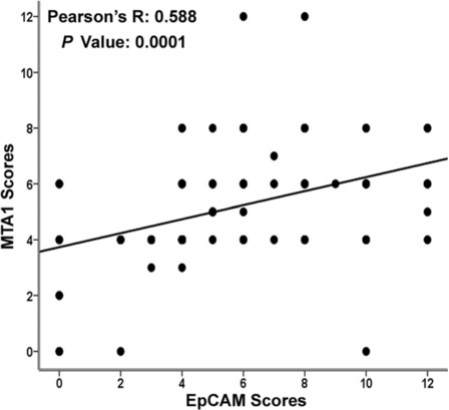
MTA1 expression positively correlates with EpCAM expression in lung cancer patients. Statistics were calculated using Spearman testing. *, *p* < 0.01

Figure 3 showed an correlation between MTA1 and EpCAM by IHC staining in lung cancer tissues (*p* < 0.01, spearman *r* = 0.588). In detail, high level MTA1 was detected more frequently in those with EpCAM overexpression than in that with low EpCAM expression (45.8 versus 33.9 %, *p* < 0.01). We further analyzed the relationship between MTA1 and EpCAM expression based on histopathologic subtype stratification. As shown in Table 2, familiar positive correlation between MTA1 staining and EpCAM staining was found in ADC (*p* < 0.05), SCC (*p* = 0.01), and SCLC (*p* = 0.01), while no correlation was found in large cell carcinoma (LCC) (*p* > 0.05).

**Table 2:** The correlation between MTA1 and EpCAM in different cancer histopathologic types

Histopathologic types			MTA1		
			Low	High	*p*Value
Squamous cell carcinoma	EpCAM	Low	17	19	0.01
		High	5	16	
Adenocarcinoma	EpCAM	Low	9	5	0.00
		High	5	27	
Large cell carcinoma	EpCAM	Low	2	3	0.71
		High	1	2	
Small cell carcinoma	EpCAM	Low	8	1	0.01
		High	0	4	

### Univariate and multivariate survival analysis

**Fig. 4:**
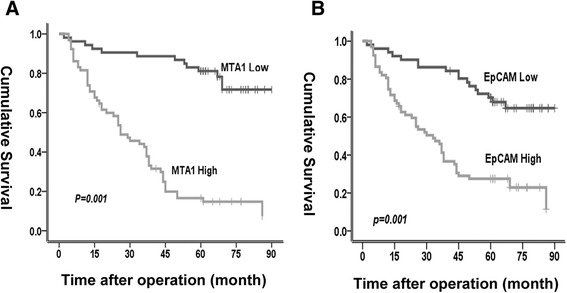
Kaplan-Meier analysis of overall survival in all lung cancer patients. **a** Patients with MTA1 overexpression had a poorer prognosis. **b** Patients with EpCAM overexpression had a poorer prognosis. The log-rank test was used to calculate *p* value. *, *p* < 0.01

According to the survival analysis, the mean overall survival of patients with MTA1 and EpCAM overexpression was 33.8 months (95 % CI: 27.4–40.3) and 38.9 months (95 % CI: 31.6–46.2) respectively, whereas those with low MTA1 and EpCAM expression was 76.6 months (95 % CI: 69.7–83.5) and 71.7 months (95 % CI: 64.1–79.3) separately (Fig. 4). In univariate analysis, tumor differentiation (*p* = 0.008), local progression (*p* = 0.001), lymph node metastasis (*p* < 0.001), clinical stage (*p* < 0.001), MTA1 and EpCAM high expression (*p* < 0.001) significantly predicted unfavorable overall survival (Table 3). Smoking status was not identified as a prognostic factor in our data analysis (*p* > 0.05), Table 3. Subsequently, all parameters with significant prognostic power in univariate analysis were included in multivariate analysis. As a result, only local progression (*p* = 0.03, hazard ratio = 2.02), differentiation (*p* = 0.03, hazard ratio = 1.52), MTA1 overexpression (*p* = 0.001, hazard ratio = 4.96) and EpCAM overexpression (*p* = 0.045, hazard ratio = 1.92) remained significant as independent prognostic factors, Table 3.

**Table 3:** Univariate and multivariate analyses of prognostic variables: Cox proportional hazards model

	Univariate	Multivariate		
Variables	*P*	*P*	Exp (B)	95 % CI for Exp (B)
Gender	0.96			
Age	0.79			
Histopathologic types	0.07			
Smoking status	0.27			
Neoadjuvant treatment	0.62			
Tumor diameter	0.008	0.86	1.04	0.66–1.66
Differentiation	0.008	0.03	1.52	1.04–2.22
Local advance	0.001	0.03	2.02	1.08–3.80
Lymph node metastasis	0.001	0.98	1.01	0.46–2.22
Clinical stage	0.001	0.84	0.92	0.42–2.02
MTA1	0.001	0.001	4.96	2.40–10.25
EpCAM	0.001	0.045	1.92	1.02–3.64

*CI* confidence interval

## Discussion

Lung cancer is one of the most aggressive malignancy, and metastasis is the leading cause of death lung cancer patients’ death. Thus, elucidating the mechanisms of lung cancer metastasis is crucial for improving the patients’ prognosis. The results presented here proved that MTA1 upregulated EpCAM in cancers for the first time, and MTA1 overexpression promoted lung cancer invasiveness in vitro. Furthermore, we demonstrated that EpCAM promoted lung cancer cells invasion and migration, and the impaired tumor cell invasion and migration abilities following MTA1-silenging can be rescued by overexpressing EpCAM. In addition, a strong positive correlation was observed between MTA1 and EpCAM in IHC data, and MTA1 and EpCAM overexpression predicted poor prognostic in lung cancer cases.

Our study has illustrated a possible correlation between MTA1 and EpCAM for the first time in ADC, SCC, and SCLC. Further we first revealed a modulation pattern between MTA1 and EpCAM. Some reports elucidating EpCAM-regulated downstream signaling molecules, such as E-cadherin and Wnt/β-catenin may help explain our founding on MTA1 and EpCAM modulation to promote tumor invasion and migration [8, 15–17]. To verify that EpCAM expression is upregulated by MTA1 in vitro, gene transfection and RNA interference were performed in lung cancer cells. We observed that EpCAM was expressed at relatively low levels in MTA1-knowdown cells, while its expression was upregulated in MTA1-overexpressing cells. Further, the suppressed metastasis ability was rescued when EpCAM was transfected to the MTA1-silenced cells, while the increased metastasis potential was inhibited when EpCAM was silenced in MTA1-overexpressing cells. These data suggested that EpCAM is a downstream molecule of MTA1 in lung cancer. Although NuRD has been reported to be a key effector in MTA1-regulated gene expression profile, we did not see modulation of EpCAM through NuRD complex (data not shown). There may be other mechanisms involved in the network.

MTA1 is known to promote the invasion and migration in a variety of cancer. In our study, we also demonstrated that MTA1 protein was significantly higher in lung cancer tissues (including small cell lung cancer) than in normal lung tissues. In addition, it was significantly positively associated with tumor diameter, clinical stage, and lymph node metastasis, suggesting MTA1 protein is engaged in the progression and aggression of lung cancer. As reported by Yu. And Li et al., our results also revealed that MTA1 overexpression independently and significantly predicts poor overall survival of the patients with early stage lung cancer [6, 18].

To date, EpCAM overexpression has been indicated to be able to predict poor prognosis of the patients with renal cancer [19], breast cancer [8], prostate cancer [20], ovarian cancer [21] and hypopharyngeal cancer [22], while its prognostic value was controversial in lung cancer. Thus, we also evaluated the correlation of EpCAM level with clinicopathological parameters and patients’ prognosis in tumor samples from lung cancer patients. Consistent with our study, reports have showed that clinical stage and lymph node metastasis of cancer patients were correlated with EpCAM expression, and EpCAM independently predicted poor overall survival in multivariate analysis [23, 24]. Contrary to our study, Pak et al. showed that lymph node metastasis was not association with EpCAM overexpression [25], what’s more, Gold et al. reported that EpCAM overexpression was a significant predictor for favorable overall survival and disease free survival [26], and even Pak et al. indicated that EpCAM overexpression was not correlated with overall survival in ADC and SCC. The difference in the reported association between EpCAM and patients’ prognosis may be caused by heterogeneity of the patient population recruited in these sutdies. For example, a collective patients’ comparison between Gold’s and ours have shown some diversity in clinical stage (16.5 versus 39.8 % in IIIA stages) and histopathologic type (61.4 versus 39.0 % in ADC, and 34.1 versus 29.6 % in SCC).

## Conclusion

In summary, our results provided a new insight into MTA1-mediated invasion and migration by enhancing EpCAM level in lung cancer. And MTA1 and EpCAM overexpression independently predicted unfavorable prognosis in our 118 lung cancer patients. Therefore, we hope that the results presented here may be helpful in the development of anti-metastatic strategies to potentially target the MTA1 alone or combined with EpCAM to inhibit the metastasis of lung cancer.
